# Supplementary material for: Use of Biological Drugs for Psoriasis: A Drug-Utilization Study Using Tuscan Administrative Databanks
Source: Int J Environ Res Public Health. 2022 Jun 2;19(11):6799. doi: 10.3390/ijerph19116799 (PMC9180218; doi:10.3390/ijerph19116799)

Table S1. Covariates: socio-demographic factors, concomitant therapies and comorbidities

Socio-demographic factors	Age, gender
Concomitant therapies	- Glucocorticoid for systemic use: ATC codes H02AB*<br>- Non-Steroidal Anti-Inflammatory Drugs (NSAIDs): ATC codes M01A*<br>- Opioid analgesics: ATC codes N02A*<br>- Small-molecule drugs for psoriasis:<br>• Acitretin, ATC code D05BB02;<br>• Antipsoriatics for topical use, ATC code D05A;<br>• Apremilast, ATC code L04AA32;<br>• Cyclosporin, ATC code L04AD01;<br>• Methotrexate, ATC code L04AX03;<br>• Psoralens for systemic use, ATC code D05BA;<br>• Psoralens for topical use, ATC code D05AD;<br>• Retinoids for treatment of psoriasis, ATC code D05BB.
Comorbidities	The following comorbidities recorded during one year before the index prescription will be evaluated:<br>- Lung disease: ICD-9 codes 490–493.99, 494, 495, 496, 500–505.99, 515–517.99; exemptions code 007*, 024*<br>- Myocardial infarction: ICD-9 codes 410–412.99<br>- Other CV disease: ICD-9 codes 394–396.99, 402, 404, 413–414.99, 424.0–424.3, 425–428.99; exemptions code A02*, B02* 021*<br>- Stroke: ICD-9 codes 430–431.99, 433–435.99, 997.02<br>- Hypertension: ICD-9 codes 401, 405; exemptions code, A31*, D31*<br>- Diabetes: ICD-9 codes 249–250.99; exemptions code 013*<br>- Fracture (of hip/spine/leg): ICD-9 codes 733.13–733.16, 733.93, 733.96–733.98, 805–806.99, 808, 820–821.99, 823, 827<br>- Depression: ICD-9 codes 296.2–296.39, 300.4, 311<br>- Gastrointestinal ulcer: ICD-9 codes 531<br>- Other gastrointestinal disorders: ICD-9 codes 532–535.7, 536.3, 536.8–536.9, 578.9<br>- Sjögren's syndrome: ICD-9 codes 710.2*<br>- Rheumatoid nodules: ICD-9 codes 446.0*<br>- Felty's syndrome: ICD-9 codes 714.1*<br>- Myopathies: ICD-9 codes 359.6*<br>- Polyneuropathy: ICD-9 codes 357.1*<br>- Cancer: ICD-9 codes 140* - 239*; ATC L01* (excluding methotrexate); exemption code 048*

Figure S1. State sequences plots of the group starting biological therapy with secukinumab, overall and by cluster

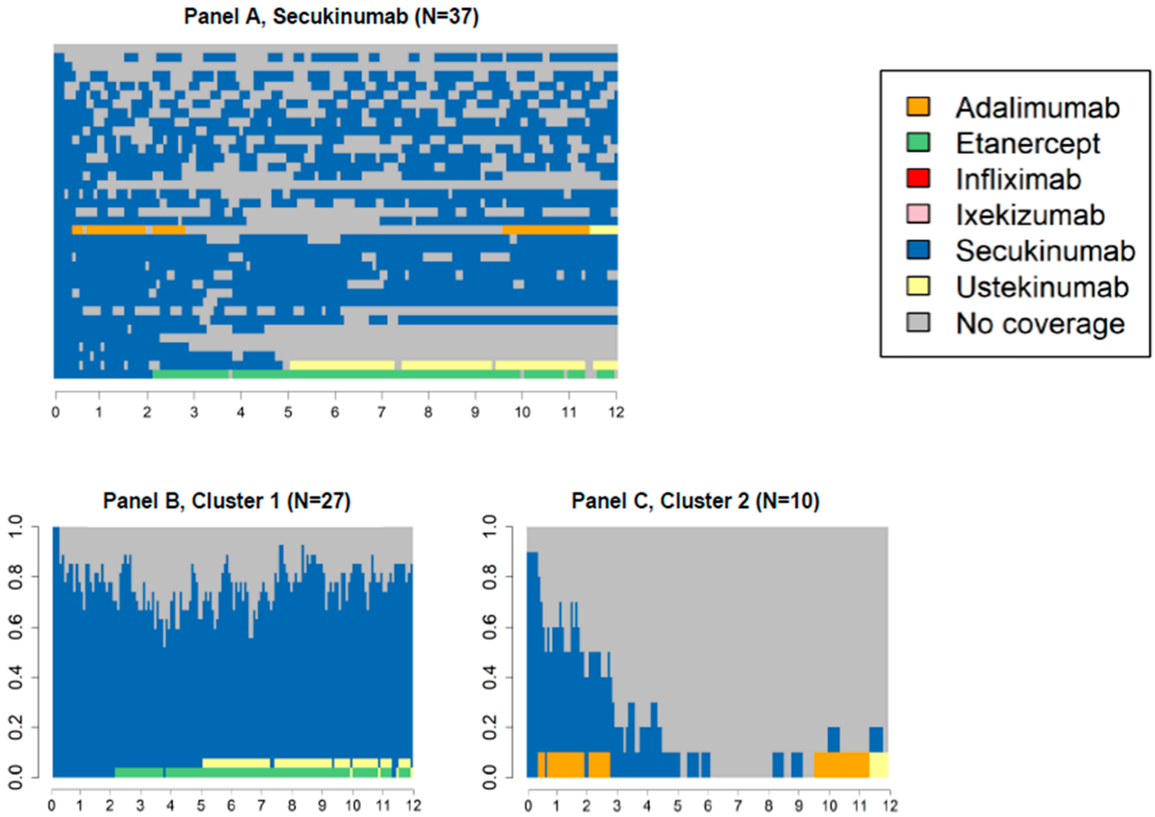

Figure S2. State sequences plots of the group starting biological therapy with ustekinumab, overall and by cluster

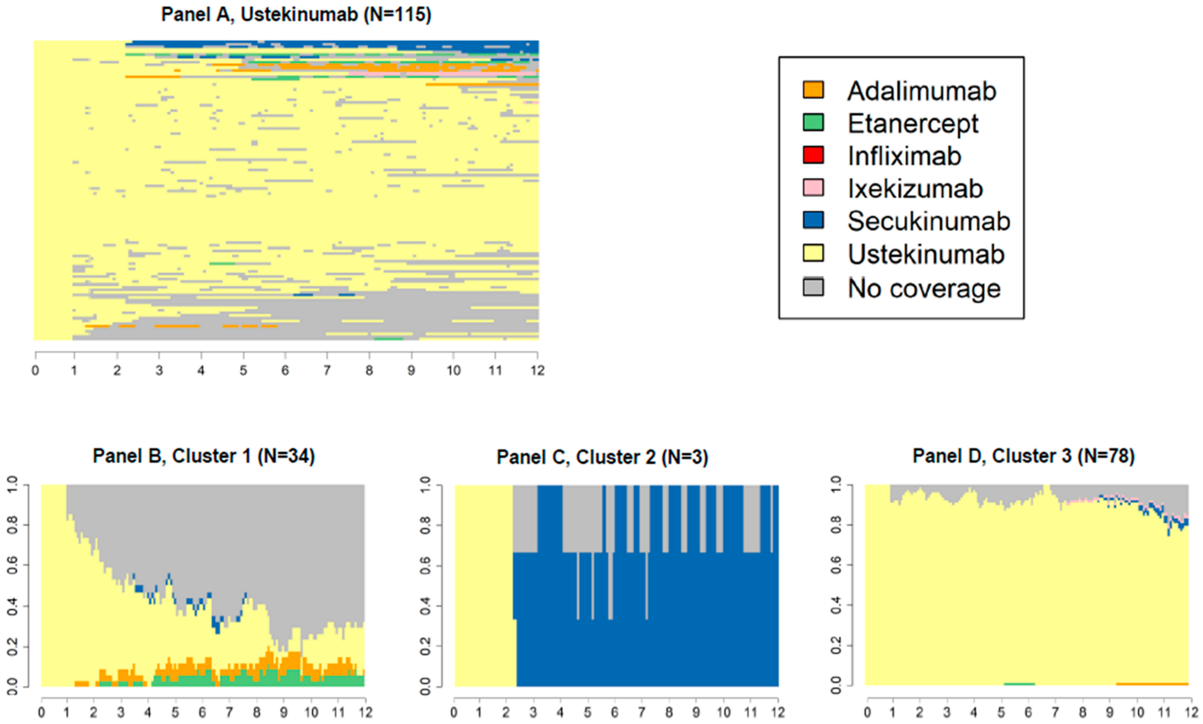

Supplement: Supplementary file 1 [file ijerph-19-06799-s001.zip › ijerph-1701916-supplementary.pdf]
